# The TIE‐93 : a cross‐cultural facial emotion recognition in French and North African Alzheimer’s dementia patients

**DOI:** 10.1002/alz.091185

**Published:** 2025-01-09

**Authors:** Renelle Bourdage, Sanne Franzen, Janne M. Papma, Juliette Palisson, Charlotte Joy, Béatrice Garcin, Pauline Narme

**Affiliations:** ^1^ Erasmus University Medical Center, Rotterdam, South Holland Netherlands; ^2^ Université de Paris Cité, Paris France; ^3^ Erasmus MC ‐ University Medical Center, Rotterdam, South Holland Netherlands; ^4^ Alzheimer Center Erasmus MC, Rotterdam, South Holland Netherlands; ^5^ Avicenne hospital, Sorbonne Paris Nord, Bobigny, Paris France; ^6^ Hôpital Avicenne, Paris, Paris France; ^7^ Université de Paris Descartes, Paris, Paris France

## Abstract

**Background:**

Facial emotion recognition testing in Alzheimer’s disease (AD) patients has been identified as key for early detection and as a marker for disease progression. Emotion recognition remains one of the most difficult domains to assess in culturally diverse populations due to a lack of culturally adapted tools. This study assessed the feasibility of a cross‐cultural test for emotion recognition, the TIE‐93, in French and North African populations living in France.

**Method:**

Forty‐six patients with AD and 148 healthy controls (aged 60 years^+^), from French and North African backgrounds (mostly Morocco and Algeria), were included in the study. The TIE‐93 is composed of eight panels with photos of actors displaying six basic emotions (happiness, anger, disgust, fear, sadness and neutral). The participant is given a daily life scenario eliciting an emotion (e.g., he is sad because is mother is very sick) and are asked to identify which of the six facial expressions displayed matches this scenario.

**Result:**

French controls (n = 82) scored higher (41 ± 5.2) than North African controls (n = 66, 36 ± 7.2) on the TIE‐93, t(146) = ‐5.64, p<.001. With French controls stopping education later in age (18 ± 4) than North African controls (13 ± 7), regression analyses were conducted to assess the contribution of education and country of origin on performance. A significant regression was found for education R^2^ = .19, F(1,146) = 34.86, p<.001, and country of origin R^2^ = .24, F(2, 145) = 23.45, p<.001. A group of education matched controls with a high education for French (n = 33) and North African (n = 33) participants were compared on test performance; no significant differences were found, t(146) = ‐1.53, p = .12. Due to small sample sizes, performance in only French AD patients (n = 37) were compared to French controls, with AD patients scoring significantly lower (31 ± 7), t(117) = ‐4.2, p<.001.

**Conclusion:**

The TIE‐93 seems promising for assessing facial emotion recognition in AD patients, demonstrating a decline in facial emotion recognition consistent with previous research literature.

